# The Impact of Obesity on the Cardiovascular System

**DOI:** 10.1155/2018/3407306

**Published:** 2018-11-04

**Authors:** Imre Csige, Dóra Ujvárosy, Zoltán Szabó, István Lőrincz, György Paragh, Mariann Harangi, Sándor Somodi

**Affiliations:** ^1^Department of Emergency Medicine, Faculty of Medicine, University of Debrecen, Hungary; ^2^Division of Metabolism, Department of Internal Medicine, Faculty of Medicine, University of Debrecen, Hungary; ^3^Division of Clinical Pharmacology, Faculty of Pharmacy, University of Debrecen, Hungary

## Abstract

Obesity is a growing health problem worldwide. It is associated with an increased cardiovascular risk on the one hand of obesity itself and on the other hand of associated medical conditions (hypertension, diabetes, insulin resistance, and sleep apnoea syndrome). Obesity has an important role in atherosclerosis and coronary artery disease. Obesity leads to structural and functional changes of the heart, which causes heart failure. The altered myocardial structure increases the risk of atrial fibrillation and sudden cardiac death. However, obesity also has a protective effect on the clinical outcome of underlying cardiovascular disease, the phenomenon called obesity paradox. The improved cardiac imaging techniques allow the early detection of altered structure and function of the heart in obese patients. In this review, we attempt to summarize the relationship between obesity and cardiovascular diseases and outline the underlying mechanisms. The demonstrated new techniques of cardiac diagnostic procedures allow for the early detection and treatment of subclinical medical conditions and, therefore, the prevention of cardiovascular events.

## 1. Introduction

Obesity has been a health problem of growing significance all over the world; its prevalence is increasing in both developed and developing countries [1]. According to WHO data, 39% of the global population above 18 years of age are overweight and of these, 13% are obese. Numerous studies have demonstrated a relationship between obesity and cardiovascular diseases (stable coronary disease, acute myocardial infarction, heart failure, cardiac arrhythmias, and sudden cardiac death). The association between obesity and hypertension, diabetes mellitus, dyslipidaemias, and sleep apnoea syndrome has also been shown to increase the incidence of cardiovascular disorders [2].

Body mass index (BMI) is used for measuring the extent of obesity; however, it gives no information on fat distribution, which is of high significance in cardiovascular risk [3]. Therefore, novel clinical measurements (e.g., abdominal circumference and the calculation of waist/hip ratio) have been introduced with the aim of characterizing central or abdominal obesity. Abdominal circumference above 102 cm in the case of men and above 88 cm in the case of women qualifies as central obesity and involves increased cardiovascular risk [4]. A waist/hip ratio above 0.9 in the case of men and above 0.85 in the case of women indicates central obesity [5].

## 2. The Relationship between Obesity and Atherosclerosis

In the past three decades, many details of the pathophysiological processes of obesity and atherosclerosis have been revealed. Previously, both diseases had been regarded as lipid storage disorders with triglyceride accumulation in the fat tissue and cholesterol esters in atherosclerotic plaques. Nowadays, both obesity and atherosclerosis are considered chronic inflammatory conditions, in which the activation of both nonspecific and adaptive immune processes is assigned a significant role [6, 7].

The pathogenesis of obesity and atherosclerosis has several common factors. In both cases, lipids, oxidized LDL particles, and free fatty acids activate the inflammatory process and trigger the disease. Inflammation is responsible for all the steps towards atherosclerosis, from early endothelial dysfunction to the atherosclerotic plaques causing complications, and is related to obesity, insulin resistance, and type 2 diabetes. The fatty tissue releases adipocytokines, which induce insulin resistance, endothelial dysfunction, hypercoagulability, and systemic inflammation, thereby facilitating the atherosclerotic process. In visceral obesity, inflammatory adipocytokines (e.g., TNF-*α*, IL-6, MCP-1, leptin, and resistin) rise to higher levels. Moreover, the increased level of C-reactive protein is associated with an increased risk of myocardial infarction, peripheral vascular disease, and diabetes mellitus [8–10]. Interestingly, a clinical study performed on obese women confirmed that body weight reduction achieved through lifestyle changes reduces the level of inflammatory biomarkers and insulin resistance. In the course of the process, adiponectin, an anti-inflammatory and insulin-sensitizing adipocytokine, is released [11]. It is important to understand the relationship between the inflammatory process and atherosclerosis and the accelerating role of obesity.

## 3. Obesity and Coronary Artery Disease

**Figure 1:**
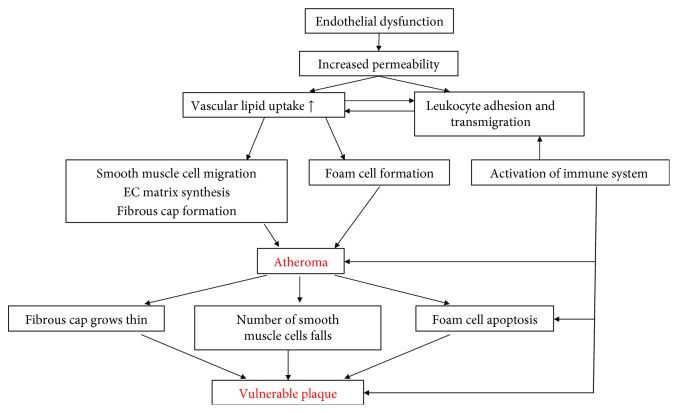
The pathomechanism of coronary artery disease in obesity.

Obesity is closely related to coronary atherosclerosis. A study performed on young patients showed that atherosclerosis begins several decades before manifested coronary artery disease. Atherosclerotic vascular lesions of patients with higher BMI values are more frequent and advanced compared to subjects with normal body weight [12]. According to longitudinal studies, at least two decades of obesity is likely to be an independent risk factor of coronary artery disease [13, 14]. A 10 kg rise in body weight increases the risk of coronary artery disease by 12% and at the same time, systolic blood pressure rises by 3 mmHg and diastolic by 2.3 mmHg as a consequence [15] (Figure 1). Furthermore, in the case of non-ST segment elevation myocardial infarction (NSTEMI) affecting young people, excess weight can be considered the most important risk factor, ahead of smoking. The higher the BMI, the sooner NSTEMI develops [16]. The same relationship can also be observed in the case of ST elevation myocardial infarction (STEMI) [17]. Based on the data available, obesity is an independent risk factor of STEMI developing at a young age [18] but at the same time excess weight can also be related to other vascular events. An increase in BMI by one unit causes a 4% rise in the risk of ischemic and a 6% rise in haemorrhagic strokes [19].

## 4. Obesity and Heart Failure

The frequency of heart failure is increasing; it is one of the major causes of death globally with a prevalence of approximately 3% in developed countries [20]. A close correlation can be observed between heart failure and obesity. According to data from the Framingham Heart Study, the rise of BMI by 1 kg/m^2^ increases the risk of heart failure by 5% in the case of men and 7% in the case of women [21]. Studies on heart failure show that 32%–49% of patients suffering from heart failure are obese and 31%–40% are overweight. In the case of obese and overweight patients, heart failure develops 10 years earlier than in the case of subjects with a normal BMI. The duration of morbid obesity is closely correlated to the development of heart failure: after 20 years of obesity, the prevalence of heart failure grows by 70% and after 30 years, the prevalence rises by 90% [22]. The significance of obesity is indicated by the fact that the Framingham Heart Study emphasized the pathogenic role of obesity for the development of heart failure in 11% of males and 14% of females [21]. The structural and functional changes of the heart observed in obesity alone contribute to a deterioration in myocardial function, which is often referred to as “obesity cardiomyopathy” [23].

Obesity leads to heart failure through several direct and indirect mechanisms. Excess weight leads to haemodynamic changes. A rise in both cardiac output and blood pressure has been observed; an increase in BMI of 5 kg/m^2^ involved a 5 mmHg rise in systolic blood pressure [24]. On one hand, it is related to the activation of the renin-angiotensin-aldosterone system and on the other hand, to the increased activity of the sympathetic nervous system [25, 26]. Obesity increases both the aldosterone level and the mineralocorticoid receptor expression, which promote interstitial cardiac fibrosis, platelet aggregation, and endothelial dysfunction. The above mechanisms explain the results of EMPHASIS-HF trial: eplerenone therapy was more beneficial for treatment of heart failure with reduced ejection fraction in patients with abdominal obesity [27, 28]. Increased blood volume facilitates venous backflow, which enhances ventricular preload causing increased ventricular wall tension and ultimately leading to ventricular dilatation. Abdominal obesity is associated with subclinical left ventricular dysfunction [29]. Hypertension increases left ventricular afterload, which raises the danger of structural and electrical myocardial remodelling. This process ultimately leads to left ventricular hypertrophy and to diastolic and later to systolic ventricular dysfunction [30].

Inflammatory cytokines (TNF-*α*, IL-1, IL-6, IL-8, etc.), whose production is increased in obesity, also play an important role in the development of heart failure [31–33]. The inflammatory mediators and acute-phase proteins in circulation cause myocardial fibrosis, which increases myocardial stiffness and may thereby lead to diastolic and later to systolic heart failure [34]. Through their effect on metabolism, tissue structure, and the extracellular matrix, leptin and adiponectin contribute directly to the myocardial transformation. Triglyceride accumulation in the cardiac muscle can regularly be observed in obese patients and facilitates the generation of toxic metabolites (e.g., ceramide and diacylglycerol), thus enhancing the apoptosis of cardiomyocytes [35–37]. The integrity of skeletal muscle mass is crucial for retaining the physical activity. Diet-induced obesity has been shown to promote muscle atrophy and catabolism. This process plays an important role in the progression of CVD in obese patients [38].

**Figure 2:**
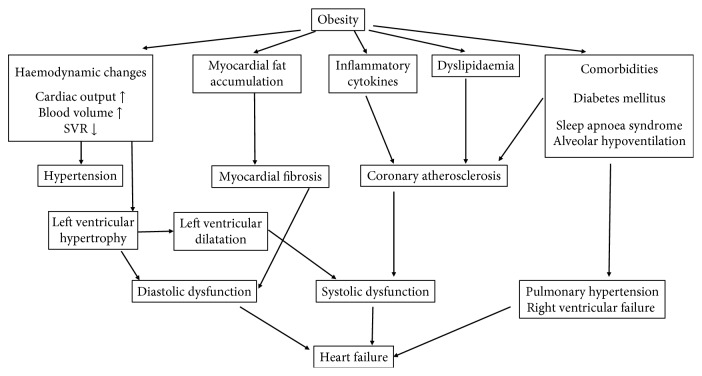
The pathomechanism of heart failure in obesity.

Moreover, obesity has been shown to increase the chances of heart failure not only by itself but also through the associated medical comorbidities. The frequently appearing insulin resistance reduces the contractility of the myocardium [39], while it enhances the activity of the renin-angiotensin-aldosterone system, which can result in hypertrophy and apoptosis of cardiac myocytes and to myocardial fibrosis [40]. Alterations in lipid metabolism enhance atherosclerosis and thereby the risk of ischemic cardiomyopathy. Unsurprisingly, obesity is therefore an independent risk factor of coronary artery disease [41]. Myocardial lipid accumulation and enhanced fibrosis can also play a pathogenic role in the genesis of various cardiac arrhythmias, which may contribute to the development of heart failure [42, 43] (Figure 2).

## 5. Obesity and Cardiac Arrhythmias

Numerous studies have demonstrated the connection between obesity and the increased risk of cardiac arrhythmias and sudden cardiac death [44, 45]. Hippocrates concluded in the 4th century already that “sudden death is more common in those who are naturally fat than in the lean.”

### 5.1. Obesity and Atrial Fibrillation

Among the cardiac arrhythmias, atrial fibrillation has the highest clinical significance. Its incidence and prevalence is increasing worldwide, affecting 1–2% of the adult population. Atrial fibrillation is responsible for about one third of the hospitalizations due to arrhythmias; it significantly increases morbidity, mortality, and health care expenditure. Over 6 million Europeans suffer from this type of arrhythmia, and this number is estimated to double in the next fifty years [46]. The occurrence of atrial fibrillation shows a correlation with age; its frequency among people aged 40–50 is under 0.5%, while it rises to 5–10% by the age of 80 years. Various studies have proven the relationship between obesity and atrial fibrillation. Obese patients have a 1.52 times higher risk for the development of atrial fibrillation compared to the normal weight population [45, 47, 48]. A 1-unit rise in BMI increases the frequency of newly developed atrial fibrillation by 4%. At the same time, in patients with atrial fibrillation, there is an increased risk for sudden cardiac death, stroke, thromboembolic complications, and heart failure. Moreover, atrial fibrillation lengthens hospitalization and worsens quality of life and physical capacity [46].

### 5.2. Structural and Electrical Remodelling Caused by Obesity

**Figure 3:**
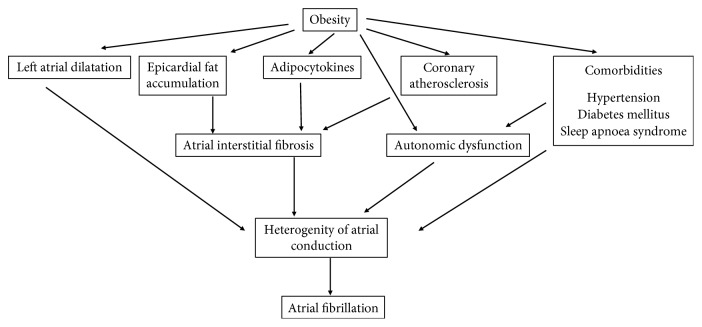
The pathomechanism of atrial fibrillation in obesity.

Obesity causes numerous anatomical and functional changes which play an important role in arrhythmogenesis. Left atrial dilation and dysfunction are known consequences of obesity. A 5 mm increase in left atrial cross diameter has been shown to raise the chances of paroxysmal atrial fibrillation 1.39 times [49]. Furthermore, recent studies have confirmed the correlation between increased epicardial fat tissue and atrial fibrillation. Through its paracrine effect, epicardial fat contributes to the development of atrial interstitial fibrosis. The increased epicardial fat, the infiltration of myocardium with adipocytes, and fibrosis together result in a heterogeneous atrial pulse conduction, e.g., anisotropy, which contributes to endo- and epicardial electrical dissociation [50–52]. All these processes facilitate the development of atrial reentry, which serves as the electrophysiological background of atrial fibrillation. Tests on both animals and humans have proven that the expansion of fatty tissue is strongly associated with insufficient capillarisation, thus myocardial hypoxia. Increased adipocyte necrosis triggers macrophage, neutrophil, and lymphocyte infiltration as well as the accumulation of proinflammatory cytokines [53] (Figure 3). It has also been proven that atrial fibrillation in obese patients shortens the refractory period of the atrial and pulmonary vein myocardial cells [54]. Enhanced adiposity triggers alterations in the ECG, too: higher amplitude *P* waves with lengthened duration, longer PR time, and *P* wave terminal force [55]. Animal research findings reveal that diet-induced obesity may be associated with prolonged atrial conduction time and heterogeneous pulse conduction. The same electrical changes were observed in the case of atrial interstitial fibrosis, increased inflammatory activity, and myocardial lipid accumulation. Remarkably, similar changes were registered in cases of congestive heart failure, hypertension, and myocardial infarction. However, the pathogenic role of complex signalisation pathways (TGF, cTGF, and endothelial system) in atrial fibrosis is not yet precisely elucidated [56].

### 5.3. The Role of Obesity and Low-Grade Inflammation in Arrhythmogenesis

The reason underlying atrial fibrillation is assumed to be the low-grade inflammation, which is mainly observed in relation to obesity. In obese patients, an increased number of leukocytes and a heightened presence of various inflammatory cytokines (C-reactive protein, interleukin-6, and tumor necrosis factor-*α*) were detected. TNF-*α* may increase the local arrhythmic vulnerability of the pulmonary vein, thereby causing atrial fibrillation [57, 58]. TGF-*β* plays an important role in the development of myocardial fibrosis [59] (Figure 3). The leptin released from adipocytes lengthens the duration of the action potential and thereby may have an arrhythmogenic effect. The roles of low-grade inflammation and oxidative stress in arrhythmogenesis have not been clearly identified yet. All the above observations are associative, so the association through inflammation remains a hypothesis only. Modifications of the ion channel function and calcium homeostasis disorder are also assumed to be underlying phenomena [60].

### 5.4. Further Arrhythmogenic Factors Associated with Obesity

In the case of atrial fibrillation, the level of atrial natriuretic peptide significantly rises and shows correlation with expected mortality [61]. Additionally, in patients suffering from atrial fibrillation, the activation of the renin-angiotensin system may be associated with atrial fibrosis and electrical remodelling [62]. Furthermore, obesity may cause autonomous nervous system dysfunction. In the case of overweight patients, excessive sympathetic activity and decreased vagal tone and consequently increased urine norepinephrine secretion and cardiac rhythm disturbances were detected [63].

## 6. Obesity and Sudden Cardiac Death

Various studies indicate a relationship between sudden cardiac death and obesity [64]. Obesity is considered an independent risk factor in the development of ventricular tachyarrhythmias. The structural remodelling in the ventricular myocardium of obese patients results in left ventricular hypertrophy and consequential systolic and diastolic ventricular dysfunctions. Myocardial hypertrophy, fibrosis, focal myocardial disarray, and increased volume of epicardial fat are also parts of the pathological process [65].

Obesity may also be associated with prolonged and inhomogeneous ventricular repolarization, which can manifest in the prolongation of the QT interval and QT interval corrected to the heart rate (QTc) measured on the 12-lead surface electrocardiogram. These ECG parameters are known as independent markers of cardiovascular mortality, and their pathological prolongation may draw attention to an increased risk of ventricular arrhythmias [66]. In the development of the pathologically prolonged and inhomogeneous repolarization observed in obesity and the electrical instability involved as a consequence, the main roles are assigned to obesity cardiomyopathy, the altered function of voltage-dependent potassium channels, and autonomic dysregulation [67, 68].

## 7. The Obesity Paradox

Even though obesity involves enhanced risk for the development of cardiovascular abnormalities, in the case of an already developed disease, excess weight and obesity are associated with a favorable prognosis. The phenomenon known as obesity paradox has been observed in the case of several cardiovascular diseases including acute and chronic heart failures [69, 70], coronary artery disease [71], acute myocardial infarction [72], hypertension, and atrial fibrillation [73, 74].

### 7.1. The Obesity Paradox and Heart Failure

In the case of patients suffering from heart failure, the findings of numerous meta-analyses have proven the phenomenon of the obesity paradox.

According to a meta-analysis processing observational studies and summarizing the data of 28,209 patients altogether, during a follow-up time of 2.7 years on the average, in the case of overweight persons with heart failure, overall mortality was 16% lower and cardiovascular mortality was 19% lower compared to the control group. The above data are even more favorable in the case of obese patients with heart failure: overall mortality rate was 33% lower and cardiovascular mortality was 40% lower compared to normal weight patients [70].

According to a more recent meta-analysis processing the data of 22,807 patients, during an average follow-up time of 2.85 years, the relative risk of overall death in the case of overweight patients with heart failure was 0.78 (confidence interval 0.68–0.89), the relative risk of cardiovascular death was 0.79 (confidence interval 0.7–0.9), and that of hospitalization was 0.92 (confidence interval 0.86–0.97) compared to normal weight patients with heart failure. At the same time, no favorable changes were observed in the case of obese patients either in cardiovascular mortality or in hospitalization; only the risk of overall mortality was lower compared to normal weight patients [75].

The analysis of Heart Failure Registry of the Heart Failure Association of the European Society of Cardiology showed inverse relationship between all-cause and cardiovascular mortality and body surface area (BSA) levels. Hospitalizations due to heart failure were not associated with BSA [76].

### 7.2. The Obesity Paradox and Coronary Revascularisation

The correlation between BMI and the outcome of clinical revascularisation was first reported in 1996, in the case of patients who had been administered balloon coronary angioplasty. According to the hospital findings, the mortality rate was higher in the case of normal weight and obese patients than in that of overweight patients [77].

Data of the multicentre BARI register processing the data of 3634 patients administered coronary revascularisation (primary coronary intervention (PCI) with catheter or surgical coronary revascularisation (CABG)) reveal that in the acute hospitalization period, there was an inverse relationship between complications and BMI only in the case of PCI-treated patients. Remarkably, in the case of the CABG group, there was an inverse relationship between mortality and BMI only 5 years after the operation [78]. It must be emphasized that clinical events following surgery, like arterial hypotension, pulmonary oedema, the deterioration of kidney function, bleeding, and mortality, were more frequent in the case of thin than in that of overweight or obese patients.

According to data of the Scottish Coronary Revascularisation Register, in the case of patients who had not suffered from coronary artery disease before and who underwent elective PCI intervention, lower mortality rate was observable in the next 5 years if their BMI value was between 27 and 30 kg/m^2^ [79].

According to data of the APPROACH register, mortality following PCI and CABG was more favorable in the case of overweight or obese persons compared to those with normal body weight [80].

On the contrary, other studies on coronary stent implantation did not support the phenomenon of the “obesity paradox.”

Interestingly, in the course of using traditional bare metal stents (BMS), an inverse relationship could be observed between BMI and clinical outcome. In the case of BMS implantation, obesity was an independent predictor of in-stent restenosis. However, a relationship between obesity and adverse events was, surprisingly, not confirmed after drug-eluting stent (DES) implantation [81].

At the same time, it is still disputed if obese coronary artery disease patients would benefit from DES implantation. According to data from the German DES.DE register, the rate of hospital complications did not differ in the cases of normal weight, overweight, or obese patients. At the time of the one-year follow-up, there was similarly no difference in mortality, myocardial infarction, target vessel revascularisation, or bleeding complications [82].

### 7.3. Explanation for the Obesity Paradox

It is well known that excess weight and obesity, as phenomena of the metabolic syndrome, lead to enhanced cardiovascular risk, endothelial dysfunction, inflammation, and atherosclerosis. The most important question is what can the explanation be for the better prognosis established in the case of overweight and obese cardiovascular patients compared to normal weight patients.

The analyses show that in the case of 2% of thin patients, comorbid conditions, mostly malignant diseases, heart failure, malnutrition, or multiple organ dysfunction, can be observed. Moreover, these patients were much older than their normal weight or obese counterparts [78, 83]. Obviously, in the case of older age patients in a generally weak condition, clinical outcomes after coronary events proved to be worse irrespective of the success of the reperfusion [84]. Advanced age and comorbid factors often result in loss of body weight [85]. In obesity, the increased level of serum lipoproteins may neutralize bacterial toxins and circulating cytokines [86]. The low level of adiponectin and the reduced catecholamine response, too, may increase the chances of survival [87]. Furthermore, in the case of obese patients, cardiovascular diseases are usually diagnosed and treated earlier than in the case of thin patients [88]. In the case of overweight and obese patients, the dose of medication required in treating the cardiovascular disease is easier to titrate considering the associated hypertension and obese patients are also more compliant with regimen than their normal weight counterparts. A possible explanation of obesity paradox is that in critical ill patients, fat which mobilized from excess adipose tissue provides energy and prevents lean tissue wasting more efficiently than exogenous nutrients [89]. In heart failure, a metabolic cardiac remodeling occurs, the fatty acid oxidation is impaired, and the glucose uptake and glycolysis are increased. The metabolic imbalance between higher energetic demand and substrate availability and lower oxidative capacity and cofactor availability (carnitine and CoA) leads to the accumulation of intermediates, which impair cardiac function, and substrates are diverged towards lipotoxic signaling pathways [90]. Alterations in mitochondrial dynamics, respiratory capacity, and ATP synthesis play an important role in the chronic cardiac energy deficit observed in heart failure [91]. Improved fatty acid utilization via dietary modification significantly ameliorates mitochondrial fragmentation and cardiac dysfunction [92].

According to more recent theories explaining the “obesity paradox,” obese patients have “larger blood vessels” and in the course of PCI, worse results are obtained in the case of patients with narrowed blood vessels [93, 94].

Antithrombotic medication is usually administered in standard doses rather than adjusted to body weight, so the dose may be too high for normal weight and thin patients, which may result in bleeding complications and this, in turn, may also contribute to higher mortality [95].

If the outcomes of cardiopulmonary stress tests are also considered, the favorable effect of higher BMI was shown to disappear [96]. According to other views, the greater muscle strength associated with a higher BMI has a favorable effect on so-called “cardiorespiratory fitness” [97, 98]. Peak oxygen consumption (VO_2_) is a positive predictor of longer survival among patients with heart failure. In multivariate analysis using VO_2_, the protective role of BMI for survival disappears [99]. The survival paradox of BMI disappears also in diabetic patients with heart failure [100]. These results support the superior prognostic power of peak oxygen consumption and diabetes compared to obesity, which attenuates the “obesity paradox” phenomenon [101].

According to the endotoxin-lipoprotein hypothesis, obese patients have higher cholesterol and lipoprotein levels, which reduce the concentration of inflammatory agents and may thus have anti-inflammatory and probably also arrhythmia protective effect. The observation that the myocardial accumulation of fat enhances the density of TNF-*α* I and II receptors, thereby facilitating the development of an antiarrhythmogenic environment, may at the same time probably serve as a kind of explanation for the development of the obesity paradox [57, 58].

## 8. Cardiac Consequences of the Haemodynamic Changes Triggered by Obesity

The volume stress associated with obesity causes both structural and functional changes in the heart. The most frequent structural changes are left ventricular hypertrophy (LVH) and left ventricular dilation, and as a consequence, diastolic, then systolic dysfunction, epicardial fat accumulation, and left atrial enlargement occur. The circulating blood volume rises; the increased cardiac output is provided mainly by the increased stroke volume and, to a lesser extent, by the increased cardiac frequency as an effect of the enhanced sympathetic tone. The above mechanisms often cause hypertension [23] (Figure 2).

## 9. Cardiology Diagnostics in Obesity

Considering the enhanced cardiovascular risk and inclination to arrhythmia observed in obesity, cardiology diagnostics are important even in the case of symptom-free obese patients. The routine 12-lead surface ECG and echocardiography are available at almost any cardiology outpatient unit nowadays.

### 9.1. Echocardiography

According to a meta-analysis published in 2014, the frequency of left ventricular hypertrophy (LVH) in obese patients is 56%; the risk of the development of LVH in the case of obese patients is 4.19 times higher than in the case of people with normal weight. Excentric hypertrophy is more frequent than the concentric type (66% vs. 34%, *p* < 0.001) [102]. LVH is usually calculated by the left ventricular mass index (LVMI), whereby it is indexed to height^2.7^. This shows a good correlation with cardiovascular mortality. Less frequently, indexation to body surface area is used [103].

**Figure 4:**
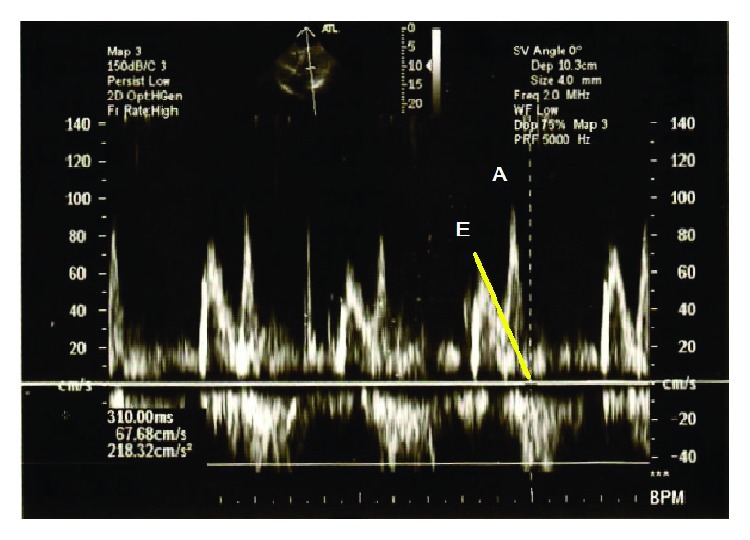
Diagnosing diastolic dysfunction with echocardiography. Transmitral flow velocities measured with pulsatile wave Doppler technique. The ratio (*E*/*A*) of the early diastolic peak velocity (*E*) and the late diastolic velocity (*A*) is lower than 1. Deceleration time (DT) is the interval from the peak of the wave *E* to its end (marked with a yellow line). In this case, its prolongation was measured (310 sec). The above alterations prove the left ventricular diastolic dysfunction (relaxation disorder).

In obesity, the prevalence of diastolic dysfunction is above 50% and shows close correlation with abdominal circumference. Among cardiovascular risk factors, age, sex, and hypertension increase the likelihood of diastolic dysfunction, which is demonstrated by what is referred to as the *E*/*A* ratio: the ratio of the mitral flow velocities measured in the early diastole (*E*) and late diastole (*A*). The value of the ratio is less than 1 in the case of diastolic dysfunction, primarily due to a rise in the peak velocity of *A*. The diagnosis also requires establishing the so-called deceleration time, i.e., the time taken from the peak to the end of the *A* wave, while the velocities of the mitral annular longitudinal movement obtained by the tissue Doppler imaging technique constitute further complementary and specification data (Figure 4). Left atrial volume enlargement is also often associated with diastolic dysfunction and can thus be considered as a marker of the latter [104]. At the same time, conventional echocardiography is sometimes unsuitable for the early diagnosis of systolic or diastolic dysfunction as the measurable parameters may still be in the normal range.

In the past decade, new echocardiographic techniques have become available that make a yet earlier diagnosis of systolic and diastolic dysfunctions possible [105]. Color Doppler imaging detects the movement and deformity of the myocardium and is thereby able to show changes in contractility [106]. The so-called “integrated backscatter” technique is able to sense changes in the reflectivity and weakening of the myocardium. These are primarily determined by the myocardial collagen content and are also influenced by the size and microstructure of cardiac muscle cells. The technique primarily provides information on myocardial stiffness, contractility, and the extent of fibrosis, in a noninvasive way [107, 108]. Pulsatile wave tissue Doppler imaging (PW-TDI) measures the cardiac muscle movement velocity. These Doppler parameters are more precise and easier to reproduce than those obtained by means of 2D, M-mode echocardiography [109]. 3D imaging has lately been introduced in cardiology as well, which makes the determination of the ejection fraction (EF) and the volume of the left atrium and the left ventricle more precise [110]. A comparison with MRI imaging proved the advantages of 3D echocardiography.

### 9.2. Electrocardiography

In case of obesity, the QT interval corrected to the heart rate (QTc) is prolonged and QT dispersion (QTd) also increases [111, 112]. These electrocardiographic differences show a correlation with an enhanced disposition to ventricular arrhythmia. In the past two decades, new markers of ventricular repolarization have been identified, which characterize cardiac muscle vulnerability in coronary artery disease, hypertrophic cardiomyopathy, and long QT syndrome very well: T peak-end (Tpe) interval, T peak-end dispersion, and Tpe/QT ratio (arrhythmogenic index) [113–115]. Studies performed on obese patients showed, however, that statistically significant prolongation compared to the control values was observed only in the case of QT interval and QTc; there were no similar differences observed in the case of the other electrocardiographic parameters [116].

Using the above testing procedures, the slight structural, electrical, and functional changes of the heart can be detected. Consequently, symptom-free patients with enhanced risk for ventricular arrhythmias can be identified at an early stage.

## 10. Summary

Excess weight and obesity are associated with an increased risk of cardiovascular diseases. This is a consequence on the one hand of obesity itself and on the other hand of associated medical conditions (hypertension, diabetes, insulin resistance, and sleep apnoea syndrome). In case of already established cardiovascular diseases, the mortality of overweight and obese patients is often lower than that of people with a normal body weight, which is known as “obesity paradox.” The exact mechanism of the latter is not clear yet. Considering the increased cardiovascular risk, the regular cardiology screening, and control of still symptom-free obese patients is important for the early diagnosis and treatment of subclinical medical conditions.
